# 5G Technology in Healthcare and Wearable Devices: A Review

**DOI:** 10.3390/s23052519

**Published:** 2023-02-24

**Authors:** Delshi Howsalya Devi, Kumutha Duraisamy, Ammar Armghan, Meshari Alsharari, Khaled Aliqab, Vishal Sorathiya, Sudipta Das, Nasr Rashid

**Affiliations:** 1 Department of AI & DS, Karpaga Vinayaga College of Engineering and Technology, Chengalpattu 603308, Tamil Nadu, India; 2 Department of Biomedical Engineering, Karpaga Vinayaga College of Engineering and Technology, Chengalpattu 603308, Tamil Nadu, India; 3 Department of Electrical Engineering, College of Engineering, Jouf University, Sakaka 72388, Saudi Arabia; 4 Faculty of Engineering and Technology, Parul Institute of Engineering and Technology, Parul University, Waghodia Road, Vadodara 391760, Gujarat, India; 5 Department of Electronics and Communication Engineering, IMPS College of Engineering and Technology, Malda 732103, West Bengal, India; 6 Department of Electrical Engineering, Faculty of Engineering, Al-Azhar University, Nasr City, Cairo 11884, Egypt

**Keywords:** 5G, wearable devices, healthcare, chronic disease, health monitoring, telemedicine, IoT, IoMT, robotic surgery

## Abstract

Wearable devices with 5G technology are currently more ingrained in our daily lives, and they will now be a part of our bodies too. The requirement for personal health monitoring and preventive disease is increasing due to the predictable dramatic increase in the number of aging people. Technologies with 5G in wearables and healthcare can intensely reduce the cost of diagnosing and preventing diseases and saving patient lives. This paper reviewed the benefits of 5G technologies, which are implemented in healthcare and wearable devices such as patient health monitoring using 5G, continuous monitoring of chronic diseases using 5G, management of preventing infectious diseases using 5G, robotic surgery using 5G, and 5G with future of wearables. It has the potential to have a direct effect on clinical decision making. This technology could improve patient rehabilitation outside of hospitals and monitor human physical activity continuously. This paper draws the conclusion that the widespread adoption of 5G technology by healthcare systems enables sick people to access specialists who would be unavailable and receive correct care more conveniently.

## 1. Introduction

The term “5G” refers to wireless communication technology’s fifth generation, which is expected to have a major impact on many aspects of modern civilization, including healthcare and wearable devices [1,2]. 5G makes use of higher bandwidth technologies such as sub-6 GHz and mmWave. 5G wireless signals are transmitted by a large number of small cell stations located in places such as light poles or building roofs. Because the millimeter-wave spectrum, the band of spectrum between 30 and 300 gigahertz that 5G relies on to generate high speeds, can only travel short distances and is susceptible to interference from weather and physical obstacles such as buildings or trees, multiple small cells are required. They have higher data transmission speeds because they use higher-frequency millimeter waves, i.e., about 100 times faster than current 4G speeds, and with extremely low latency, i.e., less than one millisecond of delay versus approximately 70 milliseconds on the 4G network. All of this functionality is achieved while simultaneously reducing energy consumption by all connected devices due to low-power supplies. Because 5G spreads at higher frequencies, “base stations” must be densely populated roughly every 250 m, and signal degradation becomes more difficult. Nonetheless, such an implementation provides a once-in-a-lifetime chance to ensure all of these technologies are delivered in real time to the point of care. 5G technologies play an abundant role in supporting healthcare and agricultural sectors with potential. Farmers can use them to centralize data for use without chemicals, and the sensor can be attached directly to fields for precise spot detection. Additionally, they help to control water supply requirements to specified areas with disease where pesticides are necessary. Mostly, 5G wearables make it very easy to identify accurate health data in the field of agriculture. Health monitoring supports farmers in decreasing the insertion of antibiotics without an agreement on the safety of the security of the food supply. 5G is connected with health care and wearable devices for the understanding of farmers to attain precise data-driven technologies without affecting water, crops, pesticides, and waste management. The essential nutrients used in agriculture are nitrogen (N), phosphorus (P), and potassium (K), commonly called fertilizer primary multipurpose nutrients. Reasons for the importance of nitrogen, phosphorus, and potassium in agriculture include (i) healthy growth, (ii) quality of harvesting plants, (iii) preventing flower drops, (iv) avoiding chemicals, (v) improving organic plants, (vi) improving soil physical, and (vii) helping to grow roots and leaves faster.

**Figure 1:**
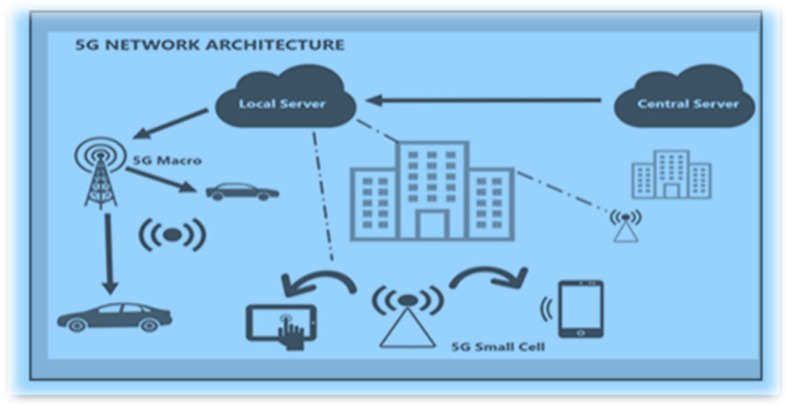
High-level 5G network architecture.

Smart health care is a critical application in 5G networks [3,4,5]. The use of 5G networks in healthcare has increased in recent years, and their applications have been supported by advancements in the internet of things, artificial intelligence, and robotics, which can change healthcare systems into a new connected ecosystem. In healthcare, Wi-Fi connectivity and 5G technology can permit patients to be observed through associated devices that continuously distribute data on key health indicators such as blood pressure and heart rate. When compared to other connectivity solutions, they provide security, increased capacity for the number of connected devices per square kilometer, greater service reliability, and mobility over in-home connectivity solutions such as Wi-Fi. This article provides an in-depth look at 5G-enabled smart healthcare and wearables. Figure 1 depicts the 5G architecture. Finally, wearable technology is an important component of future information communication technology (ICT) systems. It is still in its early stages and faces several significant challenges related to data collection and processing, security, communication and hardware limitations, user adoption, and privacy concerns. This work emphasizes them and provides readers with an extensive summary of potential methods to overcome the limitations in the current literature.

Google is one of the actors in healthcare communication. They have developed a number of smart devices for healthcare monitoring. For example, consider Google’s Fitbit tracker. It measures the oxygen level in our blood to assist us in determining where there may be an indication of significant changes in our fitness and well-being. It estimates the user’s blood oxygen saturation using optical sensors. The LEDs in the sensors have very low power and are programmed to shut down if the Fitbit device freezes or is unable to find a signal. This device incorporates privacy design principles, allowing users to track blood oxygen trends discreetly. Users must give consent in the Fitbit app to enable this feature, and they can deactivate SpO_2_ tracking on their Fitbit device at any time. Users can also access and manage their SpO_2_ data using the Fitbit data export and deletion tools in developing wearables on 5G.

In 2019, the Centers for Medicare and Medicaid Services (CMS) analyzed remote patient monitoring to improve its healthcare processing system. Additionally, the Italian Institute of Technology (IIT) and IRCSS Hospital San Raffaele established a surgical operation on 5G in Italy. Edge computing is essential for hardware and software operations inbuilt to evolve from telemedicine to real-time healthcare. Finally, Huawei discussed 11% power consumption for higher data speeds in 5G technologies for healthcare and wearables. In the future, the increase in the power consumption of 5G will consume up to 20% of the electricity in telemedicine due to the constraints of wireless communication. Technology can fail for the following reasons: infrastructure development is expensive, the issue of security and privacy has yet to be resolved, technology claims appear difficult to achieve (in the future, they may be) due to incompetent technological support in most parts of the world, and insufficient availability (especially in rural areas). The issue of health risks from mobile radiation has yet to be definitively resolved.

The remaining parts of this paper are structured as follows. The aims of this study survey are covered in Section 2. In Section 3, the review approach is discussed. Finally, Section 4 and Section 5 provide a discussion, followed by conclusions and recommendations for future work.

## 2. Review Objectives

- The primary objective of this paper is to conduct a survey of the 5G technologies that we use in healthcare and wearables.
- The secondary objective of this paper is to investigate how 5G can aid in remote patient monitoring and chronic disease management.
- The tertiary objective of this paper is to investigate how 5G can be used in IoMT and robotic surgery.

The objective of 5G is focused on wearable health monitors that can perform local data analysis without connecting to the cloud, for example, a heart rate monitor that can independently analyze health data and provide the necessary response immediately to alert caregivers when patients need help. Additionally, most researchers tabulate the frequency ranges of 5G communication technologies with their benefits and applications [1].

## 3. Review Methodology

The literature review methodology is covered in this section. Choosing the search term to use to find references is the first step in the evaluation process. The literature includes any of the following topics: 5G, wearable devices utilizing 5G, robotic surgery using 5G, IoMT, IoT in healthcare, and patient health monitoring using 5G. All of the literature used in this review was searched using online search engines such as Google, Google Scholar, and IEEE Xplore. We emphasize literature from credible publishers to be used in this review, such as IEEE, Elsevier, and Springer. The issue is that there are too many journals in the literature. We separated the references based on their usefulness and publication year (the most recent five years). The results we found included literature published in 2022 (11 papers), 2021 (9 papers), 2019 (12 papers), 2018 (6 papers), and 2017 (7 papers).

### 3.1. Patient Health Monitoring Using 5G

Several articles have described remote patient monitoring using a wireless body area network. The concept is to screen multiple vibrant sign limitations documented by various sensors mounted on the body exterior, or even fixed sensors, and to collect all motions. Before sending the footage to the doctor, it must first pass through a wearable receiver or radio communication gateway. Clinicians need automatically recorded data in order to correctly diagnose the patient’s actual condition. Wearable sensors are used to monitor patients remotely and with communication equipment, which is a critical component of the personalized healthcare concept [6,7,8].

5G network solutions may open up whole new options for action control, patient monitoring, and data analysis. For example, as a result of increased communication capacity in future 5G networks, the prevalence of cardiac sickness will skyrocket and Follow-up with patients and drug administration will be offered in unique ways. An enhanced solution for individually individualized medication modification could be another essential use. Dosages of medications are now given based on prior experience and possibly on periodic interval-based monitoring, such as insulin dosages for diabetic patients. Cardiac patients’ quality of life will improve if implantable dose reservoirs can be managed remotely. 5G will enable high-bandwidth practitioners in the healthcare field to communicate with one another and a central server, as well as between patients and a personal trusted gateway (PTG). A number of researchers have looked into employing 5G technology to monitor patients. The following summary will provide a quick overview of current 5G research for patient health monitoring. Zhang [9] and colleagues submitted a study titled “Real-Time Remote Health Monitoring System Powered by 5G MEC (Multi-Access Edge Computing)-IoT.” They built a telemedicine system based on MEC and AI for remote sickness diagnosis and healthiness monitoring. According to their findings, integrating various technologies such as computers, medicine, and telecommunication can considerably increase patient treatment efficiency while also lowering healthcare costs. Their suggested model displays greater accuracy of estimation over many categories in the ECG dataset in simulation results, allowing the entire system to deliver more efficient medical information. 

Lalita et al. [10]. suggested a four-layer design for a healthcare system based on the 5G new radio (NR) architecture that integrates the control plane and the user plane. They ran the simulation over two frequency ranges, 1 and 2, and measured the throughput and delay for various values of OFDM numerologies. They conducted a comparison of 4G and 5G, and it was determined that 5G has a 10 times lower latency than 4G and can support a far higher number of devices and deliver electronic conversation for improved healthcare facilities via 5G NR. Their discussion finalizes data exchange and makes diagnosing diseases faster and easier. Abdul and colleagues [11] gave a current review of state-of-the-art smart healthcare enabled by 5G and IoT, covering research trends, difficulties, taxonomy, and future research prospects. To begin, they presented a 5G smart healthcare architecture, as well as the necessary techniques to make it possible. Second, they introduced the 5G smart health care taxonomy and examined the new necessities and objectives for 5G smart health care. They presented a comprehensive overview of solutions for the network layer for IoT-based 5G smart congestion control; healthcare, including routing; and scheduling, which included both future research potential and recent work in the third step. Finally, they discussed some of the unresolved concerns and challenges that will face future 5G smart healthcare. 

Lakhal et al. [12]. suggested creating a wireless sensor network application that uses 5G technology for high-speed transmission to increase the safety of healthcare personnel. It comprises transferring medical data to a hospital server through the internet through various sensors mounted on the patient’s body, which measure physiological signs in real time and provide medical data useful for analytic and nursing purposes. The doctor will be able to access and examine the patient’s medical information by using a communication edge created by JEE that can receive an SMS in the event of an emergency. 

#### Continuous Monitoring of Chronic Diseases Using 5G

Routine checkups for most chronic conditions typically include monitoring to check on the disease’s progress or regress, as well as the emergence of complications. Such checks necessitate deciding what to track, when to track it, and how to alter therapy. Poor decisions in each area can lead to a loss of control, inefficient use of time, and potentially harmful treatment modifications. Monitoring is a type of measurement that is performed on a regular basis to help with the management of a chronic or recurrent condition. Clinicians, patients, or both can participate. Clinicians or patients can control monitoring and modification. People’s lifestyles are improved by continuous mobile health monitoring systems that provide medical support over large distances. If a patient becomes ill, this method allows them to communicate directly with a doctor over a vast distance. It cuts down on the amount of time and money it takes to get medical help [13,14,15,16]. To build and create continuous monitoring of chronic disease, a 5G network is combined with machine-to-machine (M2M) communications. A significant advancement in M2M communications and IoT is required to improve healthcare solutions. characteristics that could be anticipated in 5G technology.

- Peak data rates should be in the tens of gigabits per second range.
- Very minimal latency (as low as 1 ms).
- The network will be 100 times more efficient in terms of energy use.
- The network will be three times more efficient in terms of spectrum usage.

**Figure 2:**
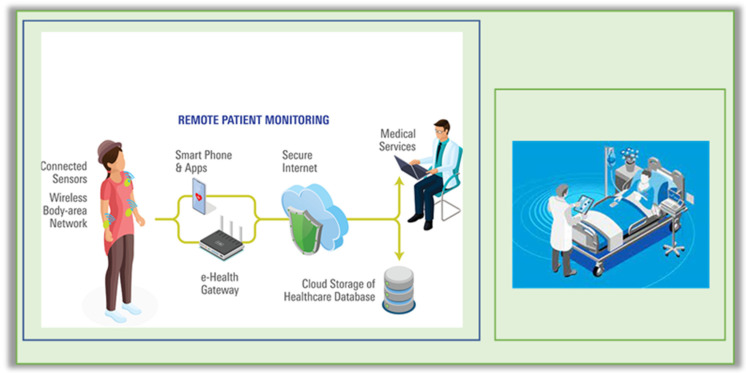
5G network for smart continuous monitoring system [19].

Various wearable gadgets, a smartphone, and a database server make up the continuous monitoring system. The smartphone is connected to the server using 5G technology in this proposed evaluation, and the architectural overview is depicted in Figure 2. The wearable sensors continuously monitor numerous physiological factors such as blood sugar, blood pressure, heart rate, and so on, and these sensors are connected to the smartphone via Bluetooth. Each smartphone has a 5G connection to the server. 

For continuous smart eHealth monitoring, Lloret et al. [17]. presented a 5G-based infrastructure and protocol. It shows how to build a sophisticated eHealth monitoring system for chronic patients. Measurements are taken on the patient’s body using wearable technology, and the data are processed by the patient’s side using a smartphone. This combines machine learning in Big Data from several hospitals, as well as patient data, to identify and make alarms. Research tests were conducted to mimic traffic flow from a large number of users to the database in order to assess the viability of 5G in our construction. Furthermore, the findings revealed that in order to continually monitor a large number of patients, the 5G network should be used since it has short delays and ensures bandwidth is available for all users. Continuous health condition monitoring was developed by Mishra and Agrawal [18] using 5G cellular channels for 24/7 sensing and transmission of physiological data. They chose pulmonary artery pressure and electrocardiogram as early physiological signals for this activity because they can aid in the diagnosis of cardiovascular diseases, the most common silent killers in modern society. It collected physiological data from a user’s body and sent it to a WBAN coordinator using a wireless body area sensor network. By partnering with the underlying sensor networks, this technology is unique in that it allows cellular system subscribers to have an online, round-the-clock health monitor with very minimal power consumption.

Wearable sensors and devices, a smartphone, and a database system that keeps all patients’ data are all part of Singh et al.’s Smart M-Health Continuous Monitoring System using 5G Technology. Artificial intelligence is used to continuously forecast a patient’s state and determine whether or not the patient needs medical intervention. If a patient becomes ill, this method allows them to communicate directly with a doctor over a vast distance. It reduces the time and cost of medical assistance. Index terms include machine to machine (M2M), abnormal data (AD), mobile health (M-Health), and artificial intelligence (AI).

The concept of mobile self-care is referred to as M-Health. It may not be as focused on clinical care and a clinical model as it is on consumers and assisting people in collecting data about themselves. For example, the AI model detects asymptomatic COVID-19 infections from cellphone cough recordings. A team of MIT researchers developed an algorithm that correctly identified people with COVID-19 based solely on the sound of their coughs. Human ears, it is claimed, cannot detect the crucial difference in the sound of the cough of an asymptomatic COVID patient. The team is working on incorporating this model into an app that, if approved by the FDA, could become a noninvasive screening tool for detecting people who may be asymptomatic. In the framework of 5G communications networks, Oleshchuk and Fensli [20] explored probable scenarios, as well as related security and privacy problems. When compared to the current situation, one of the new challenges is the possibility to have direct access to devices within the BAN. 

They recommended that for use in medicine, we need to strike a balance between patient confidentiality and privacy and the need for safety and utility, as well as ways for dealing with emergency situations. Existing authentication and authorization systems, on the other hand, were not designed with these circumstances in mind. They developed a novel method to handle emergency situations that might occur in the medical environment based on this scenario and the expansion of the RBAC model. 5GREM, a distant monitoring system for cardiovascular disease that uses an ECG biosensor as a BSN device, was introduced by Sigwele et al. [21]. This purpose is to screen and analyze the patient’s cardiac beats and lead emergency warnings to surround ambulances, caregivers, or physicians during disturbances in order to prevent heart attacks and heart failures through valid vigor. Depending on the battery level, CPU level, transmission power, delays, and task failure rate, requirements from the ECG sensor are either moved nearby on the entry, divested to adjacent mobile devices, or routed to the 5G authority. To reduce energy consumption in the mobile gateway, the 5GREM framework uses 5G technology, Mobile edge computing (MEC), and D2D task offloading. Heart attack deaths can be drastically reduced, according to 5GREM.

**Figure 3:**
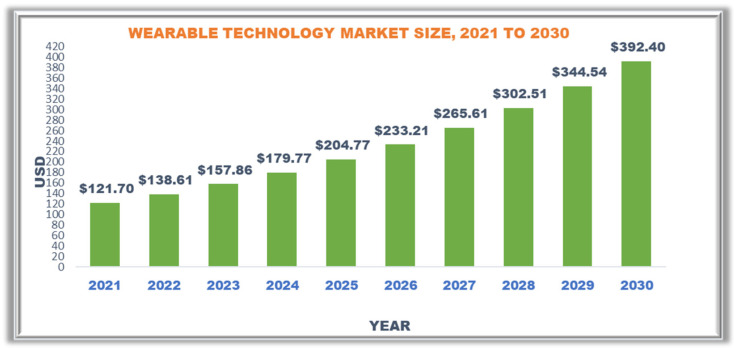
Wearable Market growth forecast.

According to Frost and Sullivan [22], 5G combined with machine intelligence, data analytics, and the internet of things (IoT) could enable new ways to tailor diagnostic tests while lowering hospital visits and medical expenditures. They looked at smartphone-based saliva testing and blood glucose self-monitoring. The report *Driving the Digital POCT Market* reveals that digital point-of-care testing (POCT) systems are gaining traction. For quick screening of respiratory disorders and saliva sample-based diagnostics, more people are opting for self-monitoring. The COVID-19 outbreak and rise in diabetic patients are fueling the digital POCT market, which addresses distant testing requirements. With the advent of 5G, machine-based intelligence, data analytics, and the internet of things (IoT), new approaches to tailoring diagnostic testing while lowering hospital visits and medical expenditures are on the horizon [23]. Figure 3 depicts a wearable market growth forecast.

### 3.2. Management of Preventing Infectious Diseases Using 5G

From a social and humanitarian standpoint, 5G communication and IoT are aimed at expanding communication systems in order to improve people’s quality of life. Control measures based on technology could be a valuable instrument in the fight against infectious disease spread. This research looks at the possibilities of upcoming technology such as 5G for regulating infection transmission, managing infectious disease prevention, and assuring health safety. Using current wireless networking solutions such as 5G, the healthcare sector can deliver a uniform, quick, and continuous service to individuals during the spread of disease. It can assist reinforce the robust communication infrastructure of the smart healthcare system in terms of greater reliability, connection stability, ultramassive accessibility, network scalability, and quick response flexibility, enabling pandemic monitoring and prevention [24,25]. Wireless communication technology can aid in the monitoring of virus spread, as well as the improvement of health, treatment, and socioeconomic sectors. In catastrophe situations, hospitals are quickly overwhelmed by the large number of people seeking treatment. Due to a lack of medical professionals and equipment, as well as a limited patient capacity, maintaining control of the situation is challenging. In fact, in pandemic scenarios, things deteriorate due to the virus’s rapid transmission capacity. Avoiding contact between two people, the person seeking treatment and the physician, is a good strategy to avoid this issue.

Alshammari et al. [26] examined the technology-driven 5G-enabled e-healthcare system during the COVID-19 pandemic and found eight main areas of application for COVID-19 control. According to the researchers, these are incorporating illness detection, travel history analysis, symptom identification, early diagnosis, transmission identification, and information access during the lockdown. The development of medical treatments and vaccines, as well as the mobility of people. It demonstrates that the majority of people obtain information without difficulty via health experts, social networking sites, and television. During the COVID-19 epidemic, around 42% of respondents reported feeling tense all or most of the time, according to the findings. In response to the COVID-19 problem, only 28.6% of respondents claimed they were tense at times, while the rest (about 30%) said they were not tense at all. Scientists needed to track the disease and try to slow its spread as the virus that causes COVID-19 began to spread from person to person in communities (community transmission). Contact tracing is also being used by scientists and public health workers to help stop the spread of COVID-19. Public health workers use this strategy to talk to people who have COVID-19 to learn about all the people they were physically close to while they were potentially able to spread the disease. These individuals are their contacts. Scientists can use this information to trace the chain of infection and learn how the disease spreads from person to person. Many other infectious diseases, such as tuberculosis and HIV, are prevented and controlled using contact tracing. 5G technology aided in the fight against COVID-19; for example, 5G-based ultrasound can help detect infected patients. Bluetooth low energy that is directly connected to a 5G network can help with contact tracing. We incorporated the frequency range, benefits, and applications of each healthcare and wearables device on 5G in this paper, and it is mentioned in Table 1.

### 3.3. Robotic Surgery Using 5G

Over the past two decades, telesurgery has evolved as a medical specialty. The concept is pretty straightforward: a highly skilled surgeon operates on a patient outside of the operating theater. Two main pieces of equipment are used to carry out the surgery: a robot that is placed in the operating room, and a remote station from which the surgeon operates the robot. A dedicated internet connection is used to establish communication between the two parties. In the orthopedic field, the robot approach has only lately entered clinical practice. It enhances implant placement precision and repeatability and has a lot of potential in terms of enhancing and assuring a better and safer clinical outcome for orthopedic surgery. The interchange of medical data is at the heart of the remote surgery concept [27,28,29,30]. Medical data, including photos, audio, and video, is digitized and transmitted via cable or wireless telecommunication networks. Surgeons can utilize the networks to remotely control the surgical robot and perform operations.

Two main pieces of equipment are used to carry out the surgery: a robot that is placed in the operating room, and a remote station from which the surgeon operates the robot. A dedicated internet connection is used to establish communication between the two parties. The goal of this study was to see if utilizing a robot might help establish the efficacy and practicality of fifth-generation (5G) wireless technologies. Surgeons can use 5G connectivity to remote control a medical robot or other surgical instrument permitting them to function as if they were in the same room. For the first time, different specialists may work together utilizing 5G technology to accomplish groundbreaking surgical procedures from any place in the world. The first 5G remote brain surgery has been completed by doctors. The patient was suffering from Parkinson’s disease and was located nearly 1500 km away. The patient acknowledged a deep brain stimulus implant through a three-hour operation. Dr. Zhipei accomplished the ground breach operation from his Sanya City place, using a computer powered by China Mobile and Huawei’s 5G network to manipulate equipment in Beijing.

**Figure 4:**
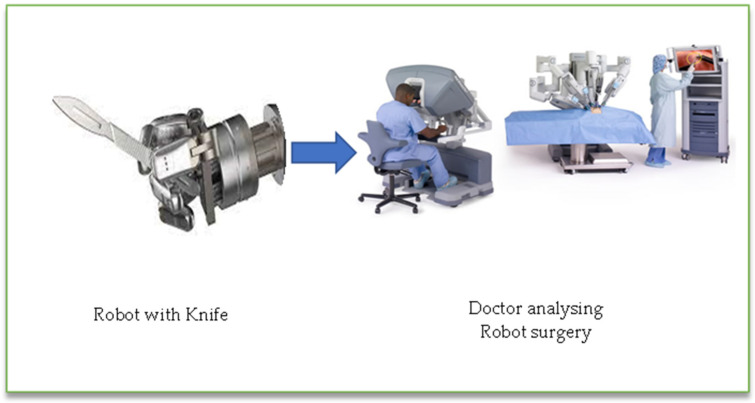
Involvement of robots in surgery.

Robotic surgery care could benefit from 5G networks. 5G networks will be beneficial to video calls, gaming, and download speeds [31,32,33,34,35,36]. On the other hand, specialized surgeons anticipate a growth in global, robotic-assisted long-distance surgery. Telepresence and telesurgery both benefit from the benefits of a 5G connection. On the other hand, the promise of 5G goes beyond just increasing the speed of today’s technologies. Surgeons will be able to integrate medical devices and collect data in previously unimagined ways as a result of the greater volume of data that can be analyzed at rapid rates. For the first time, experts from all over the world could work together. It has the potential to reduce operating expenses while also increasing the quality of care for patients in rural areas in the long run [37,38,39,40,41]. Assistive and fully automated robotic surgery are the two main types of robotic surgery. Doctors use assistive equipment to make small incisions, assure proper placement of medical devices, and close up patients following surgery. Fully automated surgical equipment can perform end-to-end procedures without the need for human intervention. Work on completely autonomous surgical robots is now ongoing, according to the IEEE. In 2016, the Smart Tissue Autonomous Robot (STAR) was able to repair a pig’s intestine on its own, with far higher precision than a human surgeon [42]. Fujian Medical University, Huawei; China Unicom Fujian Branch Mengchao Hepatobiliary Hospital; and Suzhou Kangduo Robot Co., Ltd., conducted the world’s first 5G surgery animal trial [42]. The procedure takes about 60 min to complete, with very little downtime. There was no blood spilled during the procedure, and the surgical wounds were clean. After surgery, the experimental animals’ vital signs remained constant. The operation was able to reach this goal thanks to Huawei’s 5G net technology’s enormous bandwidth, low latency, and large connection capabilities. The 5G network handles the control connection, as well as the two-video links at both tops of the remote-control machine. “High-definition video has attained the same experience as a fiber-optic line based on 5G network control experience.” The surgeon gave a favorable endorsement. Remote diagnosis, remote care, and remote surgery will be gradually used as 5G medical association applications mature, effectively increasing the value of the lifetime of sick persons. Telerobotic spinal surgery using a 5G network was proposed by Tian et al. The goal of their research was to see if fifth-generation wireless networks spinal telerobotic surgery was effective and feasible in our first 12 patients [43]. They came to the conclusion that 5G telerobotic spinal surgery is both efficacious and practicable for the safe care of spinal ailments, and 5G remote robot-assisted spinal surgery is accurate and dependable. They also came to the conclusion that 5G-based telerobotic spinal surgery is accurate, safe, and dependable. Robotics in spine surgery is a newer technology that has a lot of potential in the future. Surgical precision with robotically inserted instruments appears to be high. The influence of robotics on radiation exposure, on the other hand, is unclear and appears to vary depending on approach and robot type. Fifth-generation (5G) network implementation is currently underway in numerous nations throughout the world. The development of innovative technology has allowed medical professionals to operate on cadavers, human patients, and animal models remotely and through telemedicine. To inform readers about the innovative technology, and viability, demonstrate its efficacy, and highlight potential advantages for cancer patients who need surgical interventions, the authors of the following article go into great detail about the history of robotic surgical systems, a summary of the internet and 5G networks, and the current scenario of remote procedures using the 5G network. Figure 4 shows how robots are involved in surgery [44].

### 3.4. 5G and the Future of Wearables

The ultralow latency of 5G might expand wearables’ capabilities beyond health and wellness in the coming years, bringing new levels of utility to applications that require a remote uplink. Ambient assisted living provided by 5G-powered wearables could provide an effective alternative to live-in care for the elderly. A 5G-enabled wearable that may alert family members or healthcare experts to a dip in blood pressure or an untaken dose of medication could benefit patients’ quality of life and caregivers’ general peace of mind [45,46,47,48,49,50]. In smart homes, wearables may also provide more tailored experiences. Beyond smart locks and learning thermostats, 5G promises to relieve developers from speed constraints and compatibility difficulties, allowing users to enjoy more ambient and intuitive connectivity. It might also pave the way for the next AI-powered virtual assistant—one that caters to every member of the family and gives what they require without prompting. Wearables have the potential to evolve from handy additions to indispensable tools that help people learn more about themselves and their surroundings thanks to 5G. However, for wearables, speed is not the most appealing aspect of 5G. For starters, 5G has significantly reduced latency than 4G. Data transfer time between two places decreases from roughly 20 milliseconds to around 1 millisecond. For most use scenarios, that is near-instant, which is critical for an autonomous car that has to know if its route needs to change right away [51,52,53,54]. The following important categories can be used to categorize major 5G disruptions:

- Device-centric architectures;
- Wearable antennas;
- Massive multiple input/multiple outputs (MIMO);
- Smart devices and the IoT;
- The mmW band.

#### 3.4.1. Pros of Wearable Technology in Healthcare

##### Remote Health Monitoring and Early Diagnosis

By using wearable technology in healthcare, it is possible to track a patient’s health statistics and identify patterns using machine learning and AI. The information gathered about each person is then utilized to anticipate possible health issues before they arise, enabling the deployment of cheaper and more efficient preventive interventions as opposed to treating a disease once it has already taken hold [55,56,57,58,59].

#### 3.4.2. Cons of Wearable Technology in Healthcare

##### Sensing Physiological Parameters

Wearable medical devices that are affixed to the human body track biological and physiological data while detecting and monitoring changes. The problem arises from inaccurate and inconsistent measurements obtained during data collection, which is primarily due to a design error or improper use of the wearable. Through smart product design and solid product documentation, this problem can be easily avoided.

##### Batteries

Wearable technology typically uses a lot of power, which limits its use and consequently its advantages. Thanks to engineers’ and electronic product designers’ inventiveness, wearable device battery restrictions have thankfully improved with time.

##### Security

One of the major challenges for wearables is security, and connected medical device cyberattacks are on the rise. By adhering to Food and Drug Administration (FDA) and other security regulations, medical equipment can be made foolproof. To successfully execute these stringent regulations, businesses that produce wearable technology for the healthcare industry need to hire internal IoT engineers or a trustworthy outside consultant.

##### High Potential Cost

Despite the fact that wearables are becoming more affordable every year, hospitals and clinics may find themselves having to spend more money on new technology infrastructures and systems if they are unable to accommodate the wearable devices that patients utilize.

### 3.5. Internet of Things in 5G Communications

The internet of things (IoT) and 5G technologies are more than fair a new group of wireless technology. This is owed to the fact that 5G networks will expand the performance and reliability of these linked devices suggestively. 5G will drive origination across many industries and offer a platform for developing technologies such as the internet of things to become embedded into our economy and way of life. The keystone for solving the full capacity of the internet of things is 5G. Today, connectivity accounts for the great majority of operator IoT income, but in the next five years, revenue will also come from apps, services, and service enablement platforms [60,61,62,63,64,65,66,67,68,69]. Thales provides a variety of 5G solutions, IoT gateways, modem cards, ranging from Cinterion IoT Modules to 5G SIMs, and IoT projects that connect and secure next-generation devices while facilitating simple migration to new networks and structures. Beyond an increase in speed, 5G networks will be more reliable, leading to more reliable connections. For any internet of things, the importance of having a reliable and stable network state is especially important for connected devices such as cameras, locks, and other monitoring systems that depend on real-time updates. IoT devices will be dependent on the next-generation network’s extremely low latency, expanded coverage, and high-speed connectivity. In order for manufacturers to benefit from these developments, they must first participate in 5G-compatible products. 5G connects more devices at faster rates and practically eliminates lag, which is ideal for IoT-enabled devices. The term “mobile IoT” refers to cellular low-power wide-area systems that operate on licensed range bands. Both 3GPP narrowband IoT and long-term evolution machine-type communications are key components of the impending 5G era of smart communications. Today’s 5G networks can support mobile IoT solutions for smart logistics, smart utilities, and smart cities [70,71,72,73,74,75]. The earliest 5G applications and customer premises equipment included fixed wireless access, mobile computing, video broadcasting,

- Facilities will be able to communicate crucial upgrades to whole networks using 5G IoT without having to interrupt operations, overload servers, or freeze functionality.
- Since personal applications drastically change how we work and live, 5G IoT will improve ordinary users’ quality of life.
- Some of the recent industries that will stay to profit from 5G IoT developments include:
  - Smart utilities;
  - Agriculture;
  - Smart cities;
  - Security and surveillance;
  - Smart buildings;
  - Healthcare;
  - Smart factories;
  - Automotive and transportation.

#### 5G and Business IoT

Not only is the internet of things expected to facilitate technological advancement, but it is also estimated to sustain 22 million employees globally. The digitization of transportation, manufacturing, agriculture, and other physical industries is likely to drive this job development. Consider mines, oil derricks, building sites, and freighter fleets. Because of the time-sensitive nature of their output, these productions would yield enormously from ultrafast data transfer. We are using the internet of things in all wearables. So, we included what is the use of the internet of things in 5G and how it can help to connect wearables for monitoring patients. When it comes to IoT and healthcare, several well-known companies are at the forefront. These firms are competing for a large slice of the pie by developing products for specific medical applications, expanding collaborative research and development, and acquiring new startups. For example, the Apple Watch continues to advance its health features with each iteration, such as its FDA-approved electrocardiogram (ECG) embedded in the Series 4, and both a menstrual health-tracking feature and a dedicated research app added to the Series 5.

### 3.6. Challenges Facing Wearable Technology

Similar to any new technology, wearable computing devices go through an introduction and exploration phase before becoming mainstream. The introduction of 5G, which will offer more bandwidth and open the door to alternative solutions such as real-time health monitoring and multiple apps running at once, is a new catalyst that will quicken the life cycle of wearable technology. You can envision what kinds of applications this combination of wearables and 5G will benefit from. Clients will occasionally question the device’s purpose and utility, whereas some will flexibly discard them. Wearable technologies, on the other hand, have plentiful probability and offer far too numerous profits to be discounted. If the acceptance of smartphones is any warning, the future of wearable computing devices will be just as bright, with only our imaginings serving as a limit.

- Improvements in software architecture to make up for the challenges of using small screens for navigation.
- Wireless and local area network administration.
- Enough protection from hackers who might obtain access to the device’s data.
- Augmented reality and autonomous processing have developed a technological dependency.

Wearable devices and electronics represent a novel interface of technology and humanity, resulting in novel challenges that must account for both the technological and human aspects of the problem. Human behavior may influence the operation of wearables as much as technological advancement. The following are some of the challenges that the wearables industry is facing:

- Wearable electronics applications that are groundbreaking. The evolution of wearables has been driven by the practicality, utility, and convenience they provide since the dawn of time. The modern wearable electronics challenge is in discovering ubiquitous applications, as its future growth is dependent on emerging applications in health, wellness, and other personal needs.
- User burden reduction and integration with everyday wearables. Wearable device illustrations frequently include images of people who have been instrumented in every possible location on the body, such as the arms, legs, torso, and so on. In practice, such a scenario represents an unrealistically high user burden and is therefore unfeasible. A related challenge is the seamless integration of wearable electronics into everyday wear items such as textiles.
- Data generated by wearable devices must be interpreted in an efficient and informative manner. Wearable devices may generate a large amount of data, such as health-related sensor signals. The difficulty lies in interpreting such data streams and connecting them to health outcomes, as well as using sensor data to guide behavioral interventions and health education.
- Privacy and security. By definition, a wearable is an electronic device that resides on or close to a person and is present in a variety of life situations. The challenges include the protection of personal information, preventing the unauthorized use of wearables for biometric identification, and ownership of the data produced by wearables.

### 3.7. Next Generation of Healthcare with the IoMT and 5G

The internet of medical things (IoMT) is an important subcategory that encompasses all things linked to health care. 5G, in particular, has the potential to expand the IoMT frontier significantly. Inventors of these devices can now take advantage of the 5G data transmission speeds mandatory for real-time solutions for surgeons, as well as for connecting IoMT devices to each other and to the succeeded servers and databases that keep track of them. Furthermore, these devices may decrease the length of time a patient occupies in the hospital while also keeping clinicians more related to their patients on a regular basis, theoretically resulting in fewer hospital visits and better total care. The implementation of IoMT technology has the possibility to bring important origination to two major areas of health care: hospital practices and costs, and home care. When we are admitted to the hospital, whether it is for a reserve or a routine treatment, we all receive a plastic bracelet. It covers personal information such as our name, blood type, birth date, and so on. Your next bracelet, on the other hand, will be more like a smartwatch. Its strength is linked to your health data, your actual location in the hospital, and even screens your vibrant signs. This bracelet might also be associated with a private 5G network within the hospital, giving you the assistance of 5G connectivity while securely transferring your data. An interconnected system of medical policies, software, and health schemes and services is known as the internet of medical things (IoMT). The IoT ecosystem is finally what sets it different, despite the fact that a growing pool and general acceptance of IoT technologies are beneficial to many industries. These include a wave of sensor-based apparatuses, such as wearables and standalone methods for remote patient monitoring.

The goal of IoMT is to connect people, data, and progressions using medical devices and mobile applications to monitor patients’ health outcomes. Remote patient monitoring is the most common and useful IoMT-connected device for monitoring a patient’s health, for example, vital tracking wearables. Most of the elderly population suffers from diseases such as diabetes, cardiac ailments, and hypertension. Hence, for cardiac patients, heart monitors are vital, as they monitor arrhythmia and alert health providers about the adverse events that may take place. Regular active monitoring and heart monitoring are achieved by some consumer wearable devices such as smartwatches. Additionally, if a patient is hospitalized, IoMT platforms help the off-campus physicians and nurses to monitor senior citizens’ signs without disturbing them.

### 3.8. IoMT in the Hospital

Over time, the hospital’s IoMT apparatus may develop gradually advance. Apart from basic vital indicators, the band could also road your oxygen stages, conduct an electrocardiogram routinely, and alert nurses if you have tumbled. Your wristband might act as a nurse’s assistant, undertaking simple tests and care an eye on you [76,77,78,79]. The IoMT devices at the hospital could become more sophisticated over time, much as a smartwatch adds new abilities every year. With a combined device similar to this wristband, the amount of time saved by workers may skyrocket. Assumed that there is an approaching nursing deficiency. As a result, 5G’s skill to minimize the claim for harbors and other healthcare laborers while keeping the same level of care would be a big benefit to hospitals. By replacing resource-intensive invasive measures with noninvasive technology, important cost decreases could be realized.

### 3.9. IoMT at Home

5G’s brands are connectivity and fast data transmission, which might speed up the growth of remote care monitoring (RCM) apparatus, and being able to connect medical information in a timely and dependable way can mean the difference between life and death. Consider a patient with chronic renal disease who could profit from industrious blood monitoring to evade kidney failure. If a patient’s blood pressure or glucose levels grow and emergency care is mandatory, a 5G-connected RCM device strength sends a cautionary to both the patient and their doctor. These RCMs are previously obtainable for diabetics who involve minute-by-minute glucose specialist care. This scenario is charming gradually possible thanks to the collective strength of IoMT and 5G, and it is composed to have a significant impact on our healthcare [80,81,82,83]. This sector’s products and services advance healthcare, ease the burden on doctors and nurses, and enable patients to receive care outside of hospitals or at home [84]. IoMT comprises tracking patient prescription orders, patients’ wearable devices, and the location of patients admitted to hospitals, which can provide information to caregivers. It also includes remote patient monitoring for persons with long-term or chronic diseases. Infusion pumps that connect to analytics consoles and hospital beds with sensors that track patients’ vital signs are other medical devices that can be upgraded to or used with IoMT technology. IoMT and wearable technology are also the cornerstones of telemedicine, which has gained a lot of attention in recent years and allows patients to be monitored remotely from their homes.

**Figure 5:**
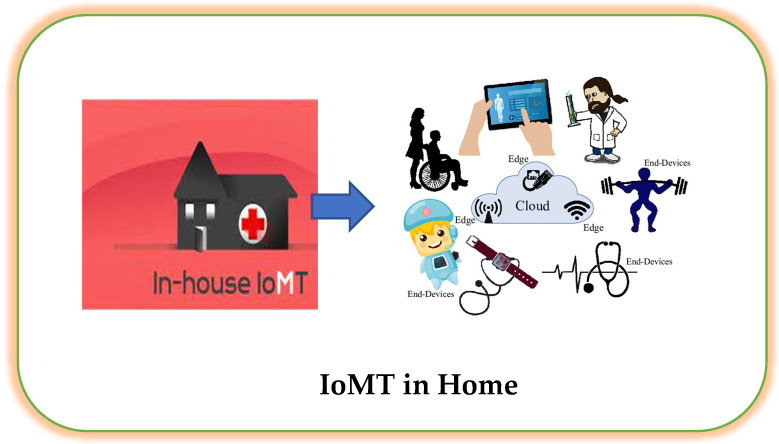
Internet of medical things at home.

A patient who receives this kind of care can avoid going to the hospital or doctor’s office if they have a medical question or a change in their condition. By establishing a connection between patients and their doctors and enabling the interchange of medical data through a secure network, it can lessen unneeded hospital visits and the strain on healthcare systems [85]. Telehealth virtual visits, personal emergency response systems, and remote patient monitoring are all included in the in-home category. A PERS blends wearable device/relay units with a live medical contact center service for the elderly who are housebound or have limited mobility to promote independence. Users can communicate and swiftly access emergency medical treatment thanks to the package. RPM includes all sensors and home monitoring equipment used to manage chronic diseases. In order to assist long-term care in a patient’s home and limit the progression of the disease, they regularly monitor physiological markers. They are also used for acute home monitoring and ongoing care of patients who have been released from the hospital to speed up recovery and avoid readmission. In order to increase adherence and outcomes, they offer customers dosing instructions and reminders for their medications. Telehealth virtual visits involve online consultations that help individuals manage their illnesses and acquire medications or recommended treatment plans. Online consultations and the assessment of symptoms or lesions under video observation and digital diagnostics are two examples [86,87,88,89]. A residential internet of things is depicted in Figure 5.

### 3.10. IoMT and 5G Innovations

**Figure 6:**
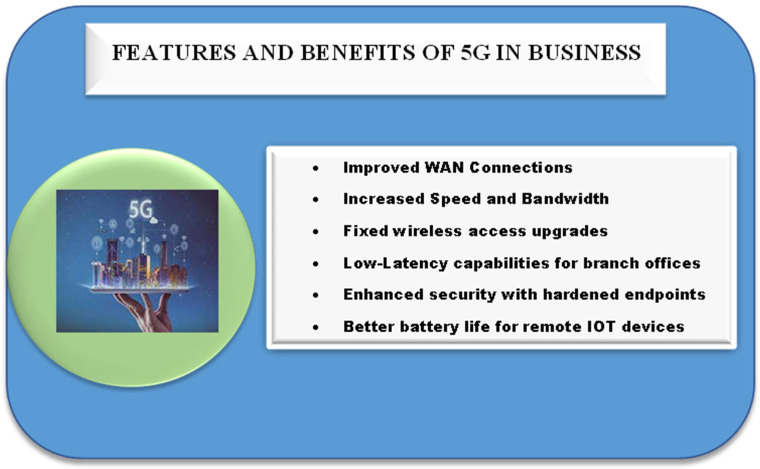
Features and benefits of 5G in business.

Before COVID-19 arrived, the IoMT was previously in overuse. Though, the coronavirus has carried even more devotion to the ways in which knowledge may recover and reorganize health care. This year’s speculation in healthcare technology has set new highs. The mainstream is now drastically reconsidering how we deliver health care, not because it needs to, but because it has to. We are grateful to travel for substitutions when some cities in our country run out of clinic cots and there is not always sufficient staff to care for patients in need. Luckily, designers and academics are already seeing how technology may improve health care, and 5G could offer the network required to alter these ideas into authenticity [90,91,92,93,94,95]. Figure 6 depicts the characteristics and benefits of 5G in business.

## 4. Discussion

**Figure 7:**
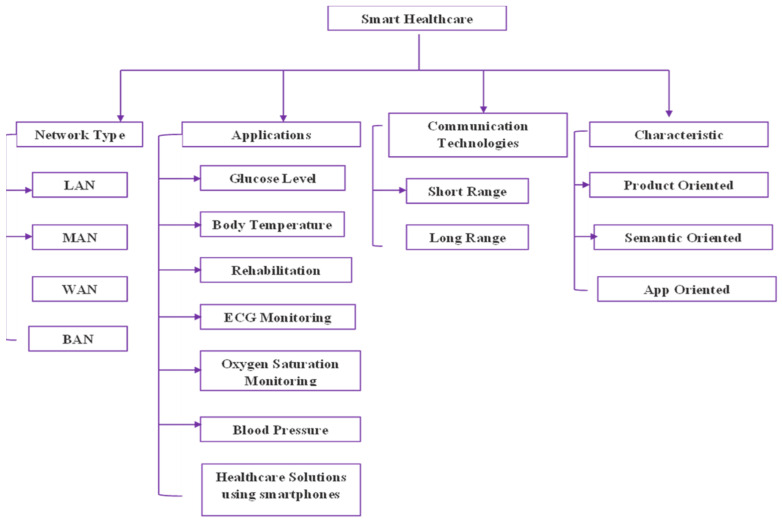
Smart healthcare and its parameters for evaluation.

This paper discussed and compared the transformative properties of 5G communications and other innovative technologies because of healthcare needs, with an emphasis on the opportunities, challenges, and limitations associated with 5G implementation and the ecosystem it has spawned. Furthermore, education, research in medicine and surgery, and clinical applications, as well as administrative infrastructure, were addressed. Furthermore, we investigated the nontechnical issues that either support or compete with this new healthcare renovation. A current scientific sign was analyzed for future trends in healthcare transformation based on the proven benefits of these innovative technologies [96,97,98]. Different communication requirements of wearable devices are summarized in Table 2. It will be possible to shorten the timeframe for patient acceptance and implementation by increasing patient awareness of these opportunities and their benefits. The communication requirements of wearable sensors and its specification are discussed in Figure 7.

## 5. Conclusions and Future Work

In smart healthcare wearable devices, fifth-generation (5G) networks will play a crucial role. From a functional and economic standpoint, and considering healthcare and wearables within the 5G network, 5G technology is crucial. We highlighted a few 5G applications in healthcare and wearables in this report. Different technological advancements for meeting these criteria in a 5G network were also examined in detail. Academics now have more possibilities for launching research projects in the area of 5G-based health-related gadgets, and making wearables smaller and lighter is a huge benefit. A sleek, fashionable band is more likely to be worn than a large, clunky watch. When smart rings or “wearables”, e.g., smart earphones, grow smaller and lighter, they become more tempting. Healthcare practitioners could utilize analytics to evaluate data sent over 5G from a wearable device for patients with chronic health conditions. The patient would receive immediate feedback on whether everything was okay, whether to schedule an appointment, or, in the worst-case scenario, whether to seek emergency help. As a result, we have seen the network “disappear” when large groups of people attempt to utilize it. 5G is designed to address these challenges and would be beneficial to the wearable technology industry. A significant amount of earlier research focused on employing AI exclusively for resource optimization, such as lowering delays and increasing reliability, or boosting overall security. The issue of identification for IoT-5G wearable medical equipment has not been solved, however. The same will be covered in our upcoming survey.

## Figures and Tables

**Table 1:** 5G frequencies with connected devices and applications.

S.No	Devices	Frequency Range	Benefits	Application
1	Healthcare	Above 6 GHz	Investigation of bioeffects	5G mobile networks
2	Healthcare	Up to 300 GHz	Avoid affecting fertility	Robust communication
3	Healthcare	30 to 300 GHz	Lower latency	Telemedicine
4	Healthcare	450 to 6000 MHz	Energy efficiency	Diagnostic in cancer
5	Wearables	sub-6 GHz	High gain and efficiency	IoT applications
6	Wearables	2.4–2.45 and 5.51–7 GHz	Minimum degradation	Wearable antenna
7	Wearables	4.4–5 GHz	93% efficiency	Wearable smart antenna
7	Wearables	200 MHz to 500 MHz	Rechargeable battery	Energy harvesting unit.

**Table 2:** Communication requirements of wearables and their specification.

Wearable Device	Specification	Requirements for Communicating Device		
		Latency	Capacity	Reliability
Smartphones	LTE, Bluetooth, mmWave Cellular and WLAN	Medium	Medium to High	Medium
Smart Watch/Glass	LTE and Bluetooth	Medium	Medium	Low
AR/VR Helmets	mmWave Cellular and WLAN	High	High	Low
Tablets	LTE, Bluetooth, mmWave Cellular and WLAN	Medium	Medium to High	Medium
Smart Clothing/Shoes	Zigbee and Bluetooth	Low	Low	Low
Medical Sensors	LTE and Bluetooth	High	Low	High

## Data Availability

The data presented in this research are available on request from the corresponding author.

## Abbreviations

**Abbreviation**	**Description**
PTG	Personal Trusted Gateway
MEC	Multi-Access Edge Computing
NR	New Radio
OFDM	Orthogonal Frequency-Division Multiplexing
IOT	Internet of Things
AI	Artificial Intelligence
JEE	Java Enterprise Edition
WBAN	Wireless Body Area Networks
M2M	Machine to Machine
M-Health	Mobile Health
AD	Abnormal Data
BAN	Body Area Network
RBAC	Role-Based access control
5GREM	5G REmote Monitoring
ECG	Electrocardiogram
MEC	Mobile Edge Computing
POCT	Point-of-Care Testing
COVID	Coronavirus Disease
STAR	Smart Tissue Autonomous Robot
MIMO	Massive Multiple Input/Multiple Output
FDA	Food and Drug Administration
3GPP	3rd-Generation Partnership Project
IoMT	Internet of Medical Things
RCM	Remote Care Monitoring
PERS	Personal Emergency Response Systems
LTE	Long-Term Evolution
RPM	Remote Patient Monitoring
